# Active inference, enactivism and the hermeneutics of social cognition

**DOI:** 10.1007/s11229-016-1269-8

**Published:** 2016-11-29

**Authors:** Shaun Gallagher, Micah Allen

**Affiliations:** 1 Department of Philosophy, University of Memphis, Clement Hall 331, Memphis, TN 38152 USA; 2 Faculty of Law, Humanities and the Arts, University of Wollongong, Wollongong, Australia; 3 Wellcome Trust Center for Neuroimaging, University College London, London, UK; 4 Institute of Cognitive Neuroscience, University College London, London, UK

**Keywords:** Predictive coding, Free energy principle, Active inference, Social cognition, Enactivism, Hermeneutics

## Abstract

We distinguish between three philosophical views on the neuroscience of predictive models: *predictive coding* (associated with internal Bayesian models and prediction error minimization), *predictive processing* (associated with radical connectionism and ‘simple’ embodiment) and *predictive engagement* (associated with enactivist approaches to cognition). We examine the concept of active inference under each model and then ask how this concept informs discussions of social cognition. In this context we consider Frith and Friston’s proposal for a neural hermeneutics, and we explore the alternative model of enactivist hermeneutics.

## Introduction

Traditional debates about social cognition have been dominated by methodological individualism, i.e., the idea that mindreading is explained by processes internal to the individual, for example, representational processes in a theory of mind [ToM] mechanism or a mirror system. Such processes are generally thought to involve feed-forward mechanisms in which abstract sensory cues (e.g., a facial expression or gesture) are recognized and mapped in a tacit meta-representational (folk psychological or simulation-based) format. Established consensus suggests there are two cortical networks responsible for our ability to understand others—a ToM network that includes the temporo-parietal junction, medial parietal cortex, and medial prefrontal cortex (e.g., Saxe et al. 2009), and mirror areas in premotor and parietal cortexes (Iacoboni et al. 2005). Taken together, the neuroscientific findings may justify a hybrid of ‘theory theory’ and simulation theory, or suggest a two-system approach of online perspective taking and offline social reasoning (Apperly and Butterfill 2009). These approaches are generally thought to be consistent with classic computational models (Stich and Nichols 2003).

In ongoing debates, however, some theorists have shifted away from classic computationalism and methodological individualism, motivated by embodied, enactivist approaches to social cognition that emphasize social interaction, the role of action and direct social perception in rich environments (e.g., Gallagher 2005, 2008a; Ratcliffe 2007; Reddy and Trevarthen 2004). On this view, the brain is part of a system of brain-body-environment, and should be understood as in some way contributing to the overall response of the organism in social or intersubjective situations (Gallagher et al. 2013) . This type of response does not entail that the brain represents the mental states of others (as maintained in traditional mindreading approaches), but that the brain participates in a more holistic and embodied process. What precisely does that mean in regard to brain function? One possible answer may be found in recent formulations of predictive models, which emphasize active inference and embodiment. On the one hand, it has been argued, the general framework of the predictive model (specifically the free-energy principle, and the concept of active inference) is consistent with autopoietic enactivist views (Bruineberg and Rietveld 2014; Bruineberg et al. 2016; Kirchhoff 2016). On the other hand, however, predictive coding’s internalist and neurocentric tendencies, as well as its concepts of ’inference’ and ’representation’, seemingly render it consistent with classic conceptions challenged by enactivist approaches.

In this paper we survey three philosophical interpretations of the neuroscience of predictive models: *predictive coding* (associated with internal Bayesian models and prediction error minimization, e.g., Hohwy 2013, 2016), *predictive processing* (associated with radical connectionism and ‘simple’ embodiment, e.g., Clark 1999, 2016) and what we propose to call *predictive engagement* (associated with enactivist approaches to cognition).^1^ We examine the concept of active inference under each model and then ask how this concept informs discussions of social cognition. In this context we consider Friston and Frith’s (2015a) proposal for a neural hermeneutics, and we explore an alternative model of enactivist hermeneutics.

## Bayesian basics

Recently there has been a resurgence of generalized predictive models of perception and neural function, which argue that the brain implements some variation of Bayes-optimal inference (Hohwy 2013; Friston and Kiebel 2009). While prediction models have a long history in neuroscience and are rooted in psychology and physiology (Helmholtz 1866; Gregory 1980; Yuille and Kersten 2006), engineering (Craik 1947, 1948), information theory (Attneave 1954; MacKay 1956), and models of receptive fields (Srinivasan et al. 1982; Rao and Ballard 1999), predictive coding models differ from the previously described approaches in emphasizing the importance and ubiquity of top-down predictions or inferences in generating perception. While predictive models had previously enjoyed some success in individual domains (e.g., motor control), this new wave emphasizes predictive processes as a universal motif which encompasses and unifies all functional domains. This ambition of the more recent expansion is now fostering intense philosophical interest and debate. In addition, through the notion of active inference the predictive approach to perception has been generalized to include an embodied role for action (Friston et al. 2011, 2013). Following from the definition of unconscious inference developed originally by Helmholtz, predictive theories view perception not as the mere passive (feed-forward) representation and manipulation of sensory information, but rather as a reciprocal process whereby generative higher-order models predict the hidden causes of sensory inputs. These models are generative in the sense that they generate predictions about the world^2^—and when statistically inverted, infer the probable causes of sensory input in terms of (posterior) beliefs. These beliefs^3^ then constrain future perception and action in an ongoing inferential loop, as constituted by an ongoing dynamical interchange between sensory information (prediction errors) and top-down predictions (prior probability distributions), as played out across the entire neuronal hierarchy.

Bayesian processing is fundamentally inferential: one tests a prior belief or hypothesis (the “prior” expressed as a probability distribution of some event or parameter) against some incoming data in order to affirm the prior or update it according to Bayes rule. Bayes rule is a simple formula from probability theory for inferring how much one should adjust one’s beliefs, in this probabilistic sense, given some overall likelihood and current evidence. Mismatches (i.e., prediction errors) between the prediction and the data are then propagated ‘forward’ in the system where they serve to further refine the original hypothesis.^4^ This process puts into effect a continual scheme of updating ‘empirical priors’ with sensory evidence.

Predictive coding is said to take place via continuous reciprocal messages passing between hierarchically arranged canonical neural circuits (Bastos et al. 2012). This view of the brain as a deep network of asymmetric connections has some empirical support both in terms of micro- and macrostructural organization (Felleman and Essen 1991; Markov et al. 2013, 2014). Predictions and prediction errors are thus ubiquitous and carry information about both the world and our activity within it. Importantly, as one moves ‘upward’ through the neural hierarchy successive levels predict increasingly more complex, multi-dimensional, contextualized information, and are, as such, sensitive to the slower timescales that are most relevant to personal phenomena. It is worth noting that this functional and temporal scalability is not a mere addition of theoretical convenience, but rather a direct statistical feature of any hierarchical Bayesian inference, and can be formally equated with computational deep learning, where the highest levels of a neural network learn complex, domain general features by predicting patterns of activity across their lower-level nodes. This equips the brain with the ability to deeply contextualize sensory information not only through some late or additive cognitive process, but also by actual interaction with the most basic levels of sensation.

**Fig. 1:**
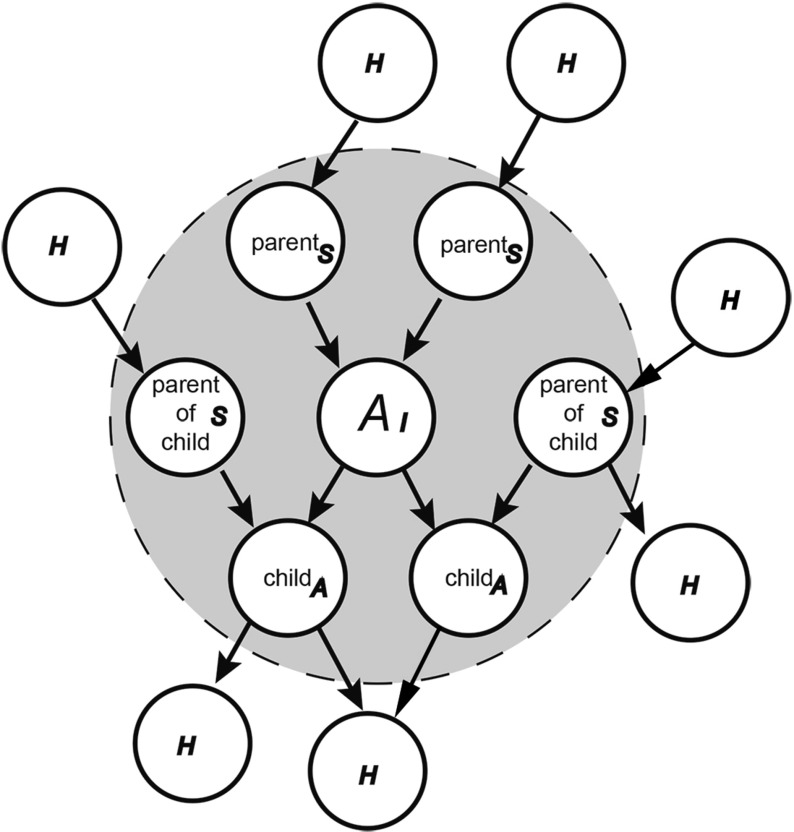
A schematic diagram of a Markov Blanket. The *circle* shaded in *gray* represents the Markov blanket of Node *A*, consisting of A, it’s children, parents, and parents of children, with parent/child being understood in terms of cause/effect. In small script, the sub-partition of internal and external states according to the Free Energy Principle (Friston 2013); *H* hidden external states, *I* internal states, *A* internal active states, *S* internal sensory states

Predictive models can also provide a more general characterization of biological systems in terms of Markov blankets, a concept derived from formal treatments of Bayesian networks, Markov decision theory, and causal dependency (Auletta 2013; Friston 2013; Friston et al. 2015). On this view, biological systems are ergodic dynamical systems that possess a Markov blanket. A system can be described as ergodic if the average time spent in a given state is the probability of being found in that state when sampled at random. It follows then, that adaptive fitness can be understood in terms of ergodicity and the endurance for survival of a system in the face of a constantly changing environment. Mathematically, this can be expressed in terms of behaviors that place an upper bound on possible states that can be occupied, such that organisms are more likely to inhabit certain states over others (namely, those that minimize their entropy or average surprise).^5^ An organism in relation to its environment is defined in terms of a Markov blanket, which is just a collection of states that define the boundary between organism and environment.^6^ The states that constitute the Markov blanket comprise sensory states and agential states that mediate action—such as movement^7^ (see Fig. 1). Markov blankets thus play the role similar to that of a cell wall, separating internal and external states to create stable (ergodic) internal dynamics that do not themselves directly impinge upon the external dynamics responsible for their emergence.

According to this characterization, external states are hidden from the internal states and can only be seen through the Markov blanket (i.e., through action). Since states external to the blanket can only be seen indirectly by those inside it (by virtue of their causal dependencies), the blanket constitutes a boundary between the world and the organism. The Markov blanket can then be sub-divided into those that are either the children of external states (sensations) or internal states (actions). Within this mathematical setup, one can formally demonstrate that if an organism is ergodic and organized along these lines, then its internal states can be described as a generative (probabilistic) model of the hidden (external) states. Thus this view deeply weds an agent’s ‘representation’ of the world to the autopoietic actions it must undertake to maintain its persistence. This can be seen when considering the dependencies between the four states (external, sensory, active, and internal) comprising the Markov blanket:

External states cause sensory states that influence—but are not influenced by—internal states, while internal states cause active states that influence—but are not influenced by—external states. Crucially, the dependencies induced by Markov blankets create a circular causality that is reminiscent of the action–perception cycle. The circular causality here means that external states cause changes in internal states, via sensory states, while the internal states couple back to the external states through active states—such that internal and external states cause each other in a reciprocal fashion. This circular causality may be a fundamental and ubiquitous causal architecture for self-organization (Friston 2013, pp. 2–3).

Thus internal states can be said to infer the hidden causes of sensory states, which are themselves influenced by the perturbation of the world by active states. The inherent circularity of this scheme means that actions (which cause changes in the external world, but not sensory states) place an upper bound on the entropy of biological states, serving to maintain a homeostatic equilibrium that is informed by internal states. According to Friston, an agent thus has two means by which to maintain its structural and functional integrity; either through the accurate internal prediction of hidden (external) causes^8^ or by acting on the environment in ways that minimize sensory surprise. Together, the ensuing changes in active (action) and internal (perception) states constitute active inference.

## An internalist predictive coding model

This characterization of predictive models is open to several interpretations. Hohwy (2013) develops an internalist version of predictive coding (PC) strongly influenced by the Helmholtzian notion of inference. On this interpretation, since the brain has no access to the external world it needs to represent that world by some internal model that it constructs by decoding sensory input—it does this by making probabilistic inferences/hypotheses about the world and correcting those inferences by reducing prediction errors. Here inference is defined as the use of Bayesian reasoning to come to a conclusion on the basis of evidence.

For Hohwy, prediction-error minimization (PEM) is primary; active inference is in service to the central processes that do the real work. He takes the concept of the Markov blanket to constitute a strict partition between world and organism, which means that the brain is cut off from the world.

PEM should make us resist conceptions of [a mind-world] relation on which the mind is in some fundamental way open or porous to the world, or on which it is in some strong sense embodied, extended or enactive. Instead, PEM reveals the mind to be inferentially secluded from the world, it seems to be more neurocentrically skull-bound than embodied or extended, and action itself is more an inferential process on sensory input than an enactive coupling with the environment. (Hohwy 2016, p. 259; see his note 14 on the Markov blanket).

On Hohwy’s reading of PC, the model of Bayesian inference entails a strong epistemic boundary that divides the brain from the rest of the body and the world (Hohwy 2013). Indeed, he identifies the boundary of the Markov blanket as explicitly located at the dorsal horn of the spinal cord, ‘where descending proprioceptive predictions from the brain are compared with ascending prediction errors from stretch receptors in the muscles’ (2016, p. 276).

Not all schemes of predictive modeling, however, are strictly internalistic; depending on how one delineates the boundaries of the Markov blanket, one can end up with a more or less enactivist, yet still prediction-oriented view of the brain (see Allen and Friston 2016). This continuum of views has implications for our understanding of social dynamics, and indeed, as we’ll now see, there is a distinct emphasis on embodiment, interaction, and narrative in some of the recent dynamical variants of predictive modeling (Friston et al. 2011; Friston and Frith 2015b).

## Predictive processing: unfolding the Markov blanket

Andy Clark’s notion of predictive processing (PP) retains some of the same PC ideas about the relation between brain and world.

[The brain] must discover information about the likely causes of impinging signals without any form of direct access to their source... [A]ll that it ‘knows’, in any direct sense, are the ways its own states (e.g., spike trains) flow and alter. In that (restricted) sense, all the system has direct access to is its own states. The world itself is thus off-limits... (Clark 2013, p. 183)

In this respect, the brain seems to be in the same position as the experimenter: The brain’s task is to take ‘patterns of neural activation and, on that basis alone, infer properties of the stimulus’ in the same way that the experimenter interprets patterns of voxels on the fMRI scan ‘to infer (decode) the properties of the stimulus that brought them about’ (Clark 2016, p. 95).

But Clark also sees PEM processes as closely tied to movement and action—i.e., active inference—and argues that PP offers support for a more embodied and enactive theory of cognition (Clark 2015, 2016).

This means that ‘inference’, as it functions in the [PP] story, is not compelled to deliver internal states that bear richly reconstructive contents. It is not there to construct an inner realm able to stand in for the full richness of the external world. Instead, it may deliver efficient, low-cost strategies whose unfolding and success depend delicately and continuously upon the structure and ongoing contributions of the external realm itself as exploited by various forms of action and intervention. (Clark 2016, p. 191).

This suggests that we do not have to think that the outcome of PP is the creation of a representation in the brain—‘a kind of internal model of the source of the signals: the world hidden behind the veil of perception’ (Clark 2013, p. 184). Rather, we can think of the brain as engaged in finding the distributed neural states ‘that best *accommodate* the current sensory barrage’ (Clark 2016, p. 192). By ‘best accommodate’, Clark denotes an approach to computation different from the classic (i.e., propositional or modular) notion. Instead, inference and ‘representation’ are here distributed across the entire network of feed-forward and feed-back connections, in an ongoing circular dynamic, as determined by a ubiquitous precision weighting mechanism. Depending on how one chooses to demarcate the boundaries of the ‘Markov Blanket’, this web of causal influence may extend to the self-active inferences in which an organism engages; inferences which are themselves a core part of the computational tapestry. Here, the brain is more like a deep network of distributed possibilities than a disembodied CPU, exploiting the dynamics of the body to achieve low-cost solutions to cognitive and environmental demands.

In active inference, the brain does this, not by sitting back and formulating hypotheses, but via ‘world-engaging action’ (2016, p. 192). For Clark, on the one hand, active inference (as in PC) is in the service of generating information that is sent back to the brain for central processing. In this respect, world-engaging action acts as a ‘complement to neural information-processing’ (Lungarella and Sporns 2005). On the other hand, Clark pushes towards a more enactivist story: problem solving is distributed across brain-body-environment, and this ‘allows the productively lazy brain to do as little as possible while still solving (or rather, while the whole embodied, environmentally located system) solves the problem’ (2016, p. 248). The enactivist story is in parentheses.

## Predictive engagement: removing the parentheses

Following the idea that active inference underscores the importance of embodiment and dynamical interaction (Friston et al. 2011; Friston and Frith 2015a, b; Kilner et al. 2007), why not remove those parentheses? On the enactivist model, we can think of the process as a kind of ongoing predictive engagement (PE)—a dynamical adjustment in which the brain, *as part of and along with the larger organism*, actively responds in ways that allow for the right kind of ongoing attunement with the environment—an environment that is physical but also social and cultural.

Conceiving of the differences or continuities among the positions of PC, PP, and PE depends on how one views the boundaries of the Markov blanket, not just where the boundaries are drawn, but the nature of the boundaries—whether they keep the world ‘off limits’, as Clark suggests, or enable coupling. For PC and PP, active inference is part of a process that produces sensory experiences that confirm or *test* my expectations; e.g., active ballistic saccades do not merely passively orient to features but actively *sample* the bits of the world that fit my expectations or resolve uncertainty (Friston et al 2012)—‘*sampling* the world in ways designed to test our hypotheses and to yield better information for the control of action itself’ (Clark 2016, p. 7; see Hohwy 2013, p. 79). On the enactivist view, however, the dynamical adjustment/attunement process that encompasses the whole of the system is not a mere *testing *or *sampling* that serves better neural prediction; active inference is more action than inference; it’s a *doing*, an enactive adjustment, a worldly engagement—with anticipatory and corrective aspects already included.

Enactivists suggest that the brain is not located at the center, conducting tests along the radiuses; it’s on the circumference, one station amongst other stations involved in the loop that also navigates through the body and environment and forms the whole. Neural accommodation occurs via constant reciprocal interaction between the brain and body, and notions of adjustment and attunement can be cashed out in terms of physical dynamical processes that involve brain and body, including autonomic and peripheral nervous systems. We can see how this enactivist interpretation can work by exploring a more basic conception operating in these predictive models, namely, the free energy principle (FEP).

## Free energy and active inference

Among the various implementations of these predictive models, the most relevant for more embodied theories of cognition are those derived from the free-energy principle, which positions the mechanisms of the predictive model within a dynamical and information theoretic treatment. Although this scheme was originally developed as a unifying theory of neural function (see, for example, Friston 2010), it has recently been expanded to serve as a bridge between information theory and biological processes themselves (Friston 2013; Friston et al. 2015). The FEP argues that biological systems are foremost defined by the tendency to resist the second law of thermodynamics, on the basis that to do otherwise would entail the unbounded increase of entropy, i.e., systemic death. This is accomplished in a manner that is analogous to autopoietic^9^ cellular processes (Varela et al. 1974), where, by avoiding unexpected states, an autonomous agent maintains its own dynamical integrity (minimizing surprise). As we will explain, these approaches are unique insofar as they attempt to make concrete linkages between notions of embodiment, environment and brain processes through the application of information theoretic principles.^10^

It is the capacity for active inference that renders the predictive model, as derived from the FEP, autopoietic and embodied. On this model an organism both generates internal dynamics (e.g., probabilistic predictions embodied in the neural network) that maximize survival (minimize free energy), and acts on the world in such a way as to cause sensory information to conform to prior predictions. Summarized simply, if an entity resists entropy and is comprised of locally interacting states or processes arranged in a Markov blanket, then FEP argues that it will survive either by accurately predicting worldly states (which entails their contextualization by the organism’s possibilities for action), or by acting on the world such as to render it unsurprising (which entails the sensitivity of action to the states of the organism). In this scheme, sensory-motor coupling is always slave to the organism’s (homeostatic) dynamics, which the system must maintain to survive. Any action or perception is constrained by this need to maintain autopoietic integrity. Heuristically, this can be thought of as the system always seeking to maximize evidence for the hypothesis that it is alive. Thus, from the basic axioms of thermodynamics, information theory, and probabilistic dependencies, the FEP proffers an explanation of how homeostatic biological systems emerge naturally from coupled dynamics between internal and external states and their co-constitution.

Following the FEP, perception and active inference are two sides of the same precision-weighted process allowing for the prioritization of actions that are likely to produce predicted outcomes and the contextualization of perception by prior action and history. This dependency of action on the structure of perception (and vice versa) provides a deeply embodied form of engagement, where the priors and actions an organism is likely to entertain are fundamentally constrained and afforded by the morphological structure of the agent’s body. In this way, the Bayesian brain is uniquely equipped to exploit the finely tuned properties of an organism’s dynamic morphological body and associated *Umwelt*.

A study by Barrett and Bar (2009); see also (Barrett and Simmons 2015; Chanes and Barrett 2016) can help to clarify how more embodied-enactivist predictive engagement can be seen as consistent with views on free energy and active inference. They propose the *affective prediction hypothesis* which ‘implies that responses signaling an object’s salience, relevance or value do not occur as a separate step after the object is identified. Instead, affective responses support vision from the very moment that visual stimulation begins’ (2009, p. 1325). Along with the earliest visual processing, the medial orbital frontal cortex is activated, initiating a train of muscular and hormonal changes throughout the body, generating ‘interoceptive sensations’ from organs, muscles, and joints associated with prior experience, which integrates with current exteroceptive sensory information. The organism as a whole responds and contributes to shaping subsequent actions.

The OFC’s ongoing integration of sensory information from the external world with that from the body indicates that conscious percepts are indeed intrinsically infused with affective value, so that the affective salience or significance of an object is not computed after the fact. As it turns out, the OFC plays a crucial role in forming the predictions that support object perception. [...T]he predictions generated during object perception carry affective value as a necessary and normal part of visual experience. (Barrett and Bar 2009, p. 1328).^11^

This suggests that priors, which include affect, are not just in the brain, but involve whole body dispositions and adjustments—‘anatomically informed priors’ (Freund et al. 2016; see also Allen and Friston 2016). In perception bodily affective changes are integrated with sensory-motor processing so that before we fully recognize an object or other person for what it or he or she is, our bodies are already configured into overall peripheral and autonomic patterns shaped by prior associations.

This implies, first, that perception is not just for recognition or identification—although many descriptions of the predictive process seem focused on simply inferring *what* the perceived object is. Nor is perception just for action—as many enactivists suggest (e.g., Noë 2004). Perception is also reward-oriented, hedonic, aesthetic, and affective in the broadest sense—and in ways that suggest that we may enjoy (and seek) perceptual surprise.^12^ Second, perceptual networks are dynamically connected to and (both in terms of those connections, and in terms of plastic changes) affected by deeply embodied processes that involve endocrine and autonomic systems.^13^ For example, fatigue and hunger involve extra-neural processes that influence brain function. Homeostatic regulation happens via mutual (largely chemical) influences between parts of the endocrine system, with some dynamical relation to the autonomic system. In cases of hypoglycemia (which may lead to slower or weaker brain function, or some brain functions turning off, or at the extreme, brain death), for example, perception is modulated because the perceptual system (brain and body) is chemically (materially) affected by hunger and fatigue. There are real physical connections here in the complex chemistry of the body-brain system in its coupling with the environment.

Brains thus participate in a system, along with all these other bodily and environmental factors, and it would work differently, because the priors and surprises in the system would be different, if these other factors were different. Enactivists often turn to dynamical systems theory to explicate such issues. DST treats organisms as complex systems composed of many individual elements embedded within, and open to, a complex environment.

As in many other complex systems in nature... parts are coordinated without an executive agent or programme that produces the organized pattern. Rather, coherence is generated solely in the relationships between the organic components and the constraints and opportunities of the environment. This self-organization means that no single element has causal priority. (Smith and Thelen 2003, pp. 343–344).

On a more dynamical and enactivist reading, the free-energy principle, which, as we’ve seen, Friston (2013) characterizes in terms of ‘circular causality’, points to a broader theoretical framework that links up with the concept of autopoiesis. A self-organizing system needs to be attuned to its ecological niche in such a way that it anticipates and minimizes surprise by taking action so that, as Bruineberg et al. (2016) put it: ‘the coupled dynamics of the organism-environment system remain within a relatively small subset of states that maintain the organism’s viability in its econiche’ (also see Friston et al. 2011). Bruineberg et al. suggest that Friston’s emphasis on active inference leaves open the possibility of the alternative enactivist interpretation, i.e., PE, which emphasizes dynamical coupling of the organism with its environment, and works out the FEP in terms of enactive autopoiesis. This may be what Friston (2013) means when he says that the agent doesn’t have a model, the agent is the model. ‘We must here understand “model” in the most inclusive sense, as combining interpretive dispositions, morphology, and neural architecture, and as implying a highly tuned “fit” between the active, embodied organism and the embedded environment’ (Friston et al 2012, p. 6).

In an autopoietic system, the boundary does not cut the system off from its environment but defines a coupling of organism-environment.

The importance of such a boundary for living organisms has been central in the autopoietic approach from the very start.... If this is the only kind of boundary that stems from the free-energy principle, then there seems to be nothing in the idea of probabilistic inference *per se *that challenges enactive cognitive science (Bruineberg et al. 2016).

This general conception can be specified in terms of defining the basic (survival-enhancing) affordances that are relative to each animal—each organism-environment. As Friston (2010, p. 127) suggests, ‘a fish out of water would be in a surprising state (both emotionally and mathematically). A fish that frequently forsook water would have high entropy’. A fish out of water can perceive and predict, and adjust their generative model all they want, but, as Bruineberg et al. suggest, it doesn’t survive unless it is afforded some action. By perception alone the organism doesn’t know or control its own viability conditions. It discovers them and can control them only by taking action. ‘So within the free-energy framework, it is *action* that does the work of actually minimizing surprisal. Actions change an organism’s relation to the environment, thereby changing the sensory states of the organism.... (Bruineberg et al. 2016). Action is not something happening in the brain, and is not just providing new sensory input for the brain; it’s what the whole organism does in its interactions with the environment, or under a different description, what a person does in the world, and this changes the world as much as it changes the brain.

On this enactivist view, the priors that inform action include bodily skills, patterns of action-readiness and affective dispositions that mesh with an affordance space. Perception is not isolated from such action; ‘perception is an inevitable consequence of active exchange with the environment’ (Friston 2009, p. 293). Bruineberg et al. rightly cite John Dewey’s notion of organism-environment. We think that Dewey’s notion of *situation* is also relevant. For Dewey (1938), the situation is not equivalent to the environment, but includes the agent in such a way that agent and environment are co-defined. The agent cannot step outside of the situation without changing it. If I am in what Dewey calls a problematic situation, I cannot strictly point to the situation because my pointing is part of the situation. My movement is a movement of the situation—and a rearrangement of objects in the situation is a rearrangement of oneself as well. As it happens, this is precisely what Gadamer (2004) calls the ‘hermeneutic situation’.

## Neural or enactivist hermeneutics of social cognition?

Chris Frith has developed his notion of ‘neural hermeneutics’ in the context of the uncertainty involved in intersubjective, face-to-face communication.

Even if I am talking with you face-to-face, I cannot access your mind to check whether my interpretation of what you have just said corresponds to what you intended me to understand. I can create a coherent story, but I can never get independent evidence about the correctness of my interpretations. (Frith and Wentzer 2013, p. 657).

Nonetheless, as Frith notes, “in spite of this apparently insurmountable difficulty,” we seem to manage to understand each other. How is that possible?

On something closer to the PC model, we can think of the uncertainty involved in our lack of access to the other person’s mind as a particular instance of the more general uncertainty tied to the brain’s lack of access to the world, e.g., when it is trying to determine whether a visual object is an apple. Thus, Friston and Frith (2015a, p. 130) suggest that ‘the criteria for evaluating and updating my interpretation of your behaviour are exactly the same criteria that underlie action and perception in general; namely, the minimisation of prediction error or (variational) free energy’. Likewise, Frith and Wentzer propose that, the same PC principle can be applied when trying to understand the mental world of others. In the social setting, however, ‘the process goes in both directions: while I am trying to understand you, you are trying to understand me’ (Frith and Wentzer 2013, p. 658).

On this view, social cognition involves processes of perception and of folk-psychological inference. The brain starts, for example, with sensory evidence of observed behaviour or spoken words; it then attempts to infer, through a series of hierarchical predictions, the thought or belief or desire that causes the behaviour.

Here the sensory evidence might be the words I hear from which I infer the idea you are trying to convey. I can test my inference, not only by predicting what else you are likely to say, but also by saying something myself and predicting how you will respond. Meanwhile you will be applying the same strategy to what I say. When our prediction errors become sufficiently low, then we have probably understood one another. (Frith and Wentzer 2013, p. 658)

Frith and Friston suggest this PC formulation captures the sense of the hermeneutic circle, understood in traditional terms of parts and whole. The whole can only be understood in terms of the parts, and the parts can only be understood in terms of the whole. ‘In the same way, in the predictive coding loop, the inferred cause (the idea, *the whole*) predicts the evidence, while, at the same time, the evidence (the words, *the parts*) modifies the inferred cause’ (Frith and Wentzer 2013, p. 658).

Frith further suggests that a simulation theory of social cognition can give us an account of the priors that allow us to get started and can address what we can call the diversity problem—i.e., the problem that others are often very different from us. Mirror neuron based simulation creates an *alignment* that purportedly solves this problem; e.g., in dyadic conversation we mimic the gestures, facial expressions, emotions, intonations and even the vocabulary of others. Even if we are somewhat different from each other, alignment ‘makes us more similar to the person we are interacting with and thereby makes motor and mental simulation more efficient’ (Frith and Wentzer 2013, p. 659).^14^ As agents enter into this seeming infinite regress of mutual prediction, it is proposed that their brains create an informational bridge of sorts, as described by Friston and Frith:

However, this infinite regress dissolves if the two brains are formally similar and each brain models the sensations caused by itself and the other as being generated in the same way. In other words, if there is a shared narrative or dynamic that both brains subscribe to, they can predict each other exactly, at least for short periods of time. This is the basic idea that we pursue in the context of active inference and predictive coding. In fact... this solution is a necessary and emergent phenomenon, when two or more (formally similar) active inference schemes are coupled to each other. Mathematically, the result of this coupling is called *generalised synchronisation* (aka synchronisation of chaos) (Friston and Frith 2015a, p. 130)

Generalised synchronization leads to the emergence of a rich tapestry of inter-relating explicit beliefs, implicit norms, and rich sensory-motor interactions.^15^ What makes social interaction unique, then, is the emergence of this unifying ‘narrative’ (generative model) and its role in shaping our own individualized perception. The Bayesian formulation thus appeals to mutually interlocking active inferences (interactions), in which the emergence of generalized synchrony allows one actor not only to predict the other, but to actively entrain their perceptual and motor states.

This is an optimistic hermeneutics. Frith and Wentzer (2013) cite Schleiermacher; and in this context they could easily have cited Dilthey on empathy (see Gallagher, in press-b). Both thinkers endorse the principle of romantic hermeneutics, namely, that we understand others ‘better than they understand themselves’. But we can also (and often do) *mis*understand the other—the possibility of which leads us to Gadamer’s notion that one doesn’t necessarily understand better—‘one understands differently when one understands at all’ (2004, p. 280). Sometimes we find ourselves in pernicious misunderstandings—as if our systems failed to produce appropriate prediction errors when they should.

In contrast to neural hermeneutics, we propose an alternative *enactivist *hermeneutics, consistent with PE, and supporting a more interactionist approach to social cognition (e.g., Gallagher 2008b). Note first that according to the interactionist approach, social cognition is not just about inferring mental states (mindreading) for purposes of predicting behavior. We see this already in the notion of alignment which points to processes that go beyond the brain—processes of interaction that depend on the other person, embodied comportment, and the particularities of the situation. Perception of another’s face, for example, activates not just the face recognition area and ventral stream, but, importantly, the dorsal visual pathway that informs our motor system—suggesting that we perceive action affordances in the face of the other (Debruille et al. 2012). That is, we don’t simply perceive the snapshot of a face in an instant, with the task of recognizing it, we *respond* dynamically over time to affordances offered by the others’ emotions as well as by their actions. Face perception presents not just objective features or patterns that we might recognize conceptually as emotions—it involves complex interactive behavioral and response patterns arising out of an active engagement with the other’s face—yielding an experience of significance or valence that shapes response.^16^ Social perception is affective in ways different from object perception. The experience of the gaze of another person directed back at you *affects* you, and your perception of the other’s emotion *affects* you, even if this affect is not consciously recognized.

Likewise, to perceive an emotion, as a bodily expression, is not simply to recognize an emotional pattern, as if the task were simply to identify the emotion (something that many experiments seem to set as the primary task). Even when presented with masked, subliminal images of angry or happy or disgusted faces or bodies, one’s autonomic and peripheral systems respond (Tamietto 2013), and such unconscious changes in arousal can influence conscious perceptual decisions (Allen et al. 2016a). Such changes in arousal are part of what the perception is, as Barrett and Bar (2009) suggest. In contexts of real interaction the issue goes beyond emotion identification; it’s rather a matter of how one affectively responds to emotion. To perceive an emotion is to experience significance and to become attuned to a valence that manifests itself as affectively relevant, and this has an effect on the perceiver’s whole body (de Gelder 2013). Just as perception is not merely the identification or recognition of an object or a pattern of features—a cold, calculative, cognitive explanation—social cognition is not just prediction and the elimination of prediction errors that seemingly finishes when the brain has inferred the correct mental state in the other’s mind—as if correctness or hypothesis confirmation were the primary goal. In real interactive situations such affective processes do not always lead to a simple or clean simulation; indeed, they can interfere with alignment and undermine simulation.^17^

In contrast to what happens in real interactions, simulation theory emphasizes inhibitory mechanisms and the sequestering or quarantining of simulated states, regarded as pretend or vicarious states (e.g. Goldman 2006). As Jacob (2008) points out, simulation is portrayed as matching what has just happened (the other’s immediate past action) and for that reason is not predictive or forward looking. Alignment, however, can be conceived, not as a mimicking of past action, but rather as an anticipatory response, a real bodily process modulated by morphologial, affective, and, as Frith might say, ‘top-top-down’ cultural factors (Roepstorff and Frith 2004; Shea et al. 2014)—priors that shape neural and extra-neural processes through plastic and metaplastic mechanisms (Malafouris 2013). That priors have an impact on the predictive dynamics of brain function means the brain has been ‘set up to be set off’ (Prinz 2004, p. 55). There is, in this regard, a hermeneutical circle that involves the brain-body-environment, and not just the working of the brain. In terms of traditional hermeneutics, it’s not Schleiermacher’s hermeneutical circle of parts-whole, resolved in a correct interpretation via the methodological bracketing or inhibition of the interpreter’s beliefs or actions, but Gadamer’s anticipatory pre-understanding that projects meaning from the perspective of the agent in the situation, which in turn leads to further interaction.

Thus, consistent with the enactivist conception of PE, the explanatory unit of perception (or cognition, or action, etc.) is not just the brain, or even two (or more) brains in the case of social cognition (cf. Hasson et al. 2012), but dynamical relations between organism and environment, or between two or more embodied agents. The enactivist thus draws the boundaries of the relevant Markov blanket not at the brain or spinal cord, but instead at the level of persons engaged in the world and with one another.^18^ The processes of social interaction include active engagements with others in socially defined environments, characterized by embodied interactions and affective processes where distinct forms of sensory-motor-interoceptive couplings are generated by the perception and response to facial expression, posture, movement, gestures, etc. in rich pragmatic and social contexts.

On the enactivist view, social cognition is an attunement process that allows an agent to perceive the other as someone to respond to or with whom one can interact. In the intersubjective context, perception serves interaction. In some cases, understanding the other person, and in some cases mutual understanding, is dynamically accomplished in the social interaction itself where some novel shared meaning (or some decision or even some misunderstanding) is instituted in a way that could not be instituted by brain processes alone inside the single individual (De Jaegher et al. 2010; Di Paolo and De Jaegher 2012). In this respect, the boundary of the Markov blanket is not drawn to enclose either the brain or the body, but is variable and extends to include relevant bits of the immediate environment, including other agents, as we engage with them. It is defined relative to the scope of engagement, which can expand to include others through the dynamics of the interaction process itself.

In a brain-body-environment system, changes or adjustments to neural processing will accompany any changes in body or environment, not because the isolated brain infers such changes and responds to them in central command mode, but because the brain is part of the larger embodied system that is coping with its changing environment. Just as the hand adjusts to the shape of the object to be grasped, so the brain adjusts to the circumstances of organism-environment. Rather than thinking of this as a kind of inference, enactivists think of it as a kind of dynamic adjustment process in which the brain, as part of and along with the larger organism, settles into the right kind of attunement with the environment. Social interaction thus involves the integration of brain processes into a complex mix of transactions that involve moving, gesturing, and engaging with the expressive bodies of others; bodies that incorporate artifacts, tools, and technologies, that are situated in various physical environments, and defined by diverse social roles and institutional practices. The priors and the surprises in the brain would be different if these other factors were different.

## Conclusion

In the contrasts between PC, PP, and PE there are not just vocabulary differences; there is a basic conceptual problem about how the different models understand brain function. These seem to be philosophical differences more than neuroscientific ones—differences that concern our understanding of concepts like representation, inference, embodiment, engagement, attunement, affordance, etc. We think that further progress on these issues will depend to some extent on sorting out the very basic issue of defining the unit of explanation (brain vs. brain-body-environment) and controversies about the vocabulary of the *explanans*. If, for example, for an enactivist PE account, inference and representation are not key parts of the *explanans*, it needs to provide further explanation of how precisely processes like adjustment, attunement, and accommodation work as part of predictive engagement.

Notwithstanding the strong contrasts, there is clearly some common ground on which the differences among predictive models can be clarified. There is much more to be said, for example, about plasticity and prior experience. The neural networks of perceptual systems and association areas may be set up by previous experience to be set off by forthcoming experience—a process captured in Jesse Prinz’s (2004) fortunate phrase of being ‘set up to be set off’. Early sensory areas are set up by prior (personal, social, and cultural) experience. For example, V1 neurons are activated not just for feature detection but in predictive anticipation of reward if they have been tuned by prior experience (Shuler and Bear 2006). In that case, priors are already part of a sensory mechanism that will directly shape perception and the organism’s response. If a scientist insists that when the activation of one set of neurons (as priors) modulates another select set of neurons (as a process of precision weighting), this just is what is meant by inference, then the enactivist can rightly ask whether one needs the vocabulary of inference to signify what is a physical modulation in this case.

Similarly, with respect to social cognition, previous embodied interaction with others attunes neural systems for further interaction (Di Paolo and De Jaegher 2012). The mirror system, for example, may be the result of prior associative processes and may activate without the requirement of inferential processes (Heyes 2010). Attunement of the mirror system through prior experience or skilled expertise with an observed action modifies perception, prediction and interpretation of that action (Calvo-Merino et al. 2005). Perception of a certain facial pattern, for example, may already be attuned to directly see that pattern as anger, or it may see it differently if the perceiver belongs to a different culture (e.g., Goh and Park 2009; Kitayama and Park 2010; Park and Kitayama 2012). In effect, individual, social and cultural factors can have a physical effect on brain processes that shape basic perceptual experience and emotional responses.

Further exploration of some of the other concepts discussed above could bring additional support to the enactivist notion of predictive engagement. Notions of circular causality in the conception of the Markov blanket (Friston 2013) are consistent with the concept of reciprocal causation in enactivist accounts of dynamical coupling; generalized synchrony also clearly relates to the notion of dynamical coupling and may be put to use in discussions of social interaction. The idea that priors may not be confined to brain states, but may be extended across embodied affective and cultural factors, is consistent with the notion that the ‘top’ of ‘top down’ modulation may extend to socially distributed practices (Roepstorff 2013; Roepstorff and Frith 2004; see Kirchhoff 2015a, b). In agreement with Bruineberg et al. (2016), and Kirchhoff (2015b) we think the FEP is in good theoretical synchrony with the enactivist conception of an adaptative autopoiesis (Di Paolo 2005; Thompson 2007; cf. Kirchhoff 2016). Accordingly, *prima facie*, there is a good case to be made for a predictive model consistent with enactivism.

## Footnotes

1 Using these terms we are making a distinction that is not made systematically in the current predictive models literature.

2 Though see below, more recent models highlight the importance of modeling the self, world, and their interaction (Quattrocki and Friston 2014).

3 It is important to note that the terms ‘inference’ and ‘belief’ as used in this context have specific technical definitions, and speak specifically to the mechanics of probabilistic inference. Beliefs are thus continuous probability distributions about some state of the world, rather than the explicit propositions and deductions more typically described in philosophy.

4 In some cases the notion of inferences in the brain is considered to be metaphorical. We find this in Helmholtz (1866): the processes of perception ‘are *like inferences* insofar as we from the observed effect on our senses arrive at an idea of the cause of this effect’; as well as in Hohwy’s conception of PC: causal inference in the brain is “*analogous* in many respects to our everyday reasoning about cause and effect, and to scientific methods of causal inference” (Hohwy 2013).

5 Note that this is just a consequence of the axiom stated previously, i.e. that any persisting biological system will be one that resists entropy.

6 Indeed, any system of causal dependencies which meets these basic requirements can be described as possessing a Markov blanket, and a biological organism could be characterized as possessing many ‘blankets-within-blankets’ (single neurons, neural networks, the embodied organism, its society, and so on).

7 Formally, the parents, children, and parents of children of a node constitute its Markov blanket, where parent/child can be understood in terms of cause and effect (see Fig. 1).

8 This is approximately equivalent to the internal inferences appealed to in classic predictive coding.

9 This is typically construed as the emergent or self-organizing persistence of an organism in virtue of its own dynamical structure.

10 Although the formalisms supporting the FEP are beyond the scope of this paper (see Feldman and Friston 2010; Friston 2010; Friston et al. 2010 for a formal treatment), we attempt to relate them here in a general fashion in order to better illuminate any potential overlap (or lack thereof) between PC, PP and PE. The FEP and its derivations depend primarily upon the assumption that biological systems are ergodic dynamical systems that possess a Markov blanket (see above).

11 It’s likely that the ventral medial prefrontal cortex (VMPFC), anterior cingulate, and insula are also involved in these processes. The VMPFC both processes value/reward and has descending modulations to the spinal cord and parasympathetic nervous system. Similarly, insula and cingulate are involved in integrating sensations from the various modalities with higher-order contexts, and they send long range modulations both to basic visual cortex, VMPFC, and the brainstem/visceral control centers (see Allen et al. 2016b).

12 We do sometimes enjoy surprises even if our systems are set up to minimize surprises. Predictive coding accounts of this are framed in pragmatic or economic terms—we trade relative short-term certainty (the possibility of a short-term loss) for the possibility of higher-degrees of long-term certainty (or gain). One may often be in a situation where an option one doesn’t know very much about will have the highest ‘epistemic’ value, because of the possibility that it could reveal entirely new options and result in the greatest long term reduction in surprise (or free energy) (Friston et al. 2016; Schwartenbeck et al. 2013).

13 Enactivists would claim that these deeply embodied processes are non-representational and that the hypothalamus operates on homeostatic principles rather than anything that can be construed as representational principles (see, e.g., Gallagher, in press-a).

14 In describing their model of generalized synchrony, Frith stays with a subpersonal simulationist account, but notes the importance of explicit social beliefs and communication: ‘we have only considered a very elemental form of communication (and implicit theory of mind). We have not considered the reflective processing that may accompany its deliberative (conscious) aspects. This is an intriguing and challenging area (particularly from the point of view of modelling) that calls on things like deceit’ (Friston and Frith 2015b, p. 12). In this regard, one question is whether the Bayesian PC account scales up to a point where one can say that the prior actually involves a theory of the folk psychological kind. Friston (2012) thinks it can. He understands motor behavior to depend on predictive coding, and he states: ‘at a more abstract level, predictions about how we will physically move are composed and generated in a way that determines how we behave. Perhaps the most important determinants of our behaviour (and their underlying predictions) are beliefs about the intentions and behaviour of others. This necessarily requires an internal model of self in relation to others and an implicit sense of agency’ (Friston 2012, p. 249).

15 For the dynamic Bayesians two systems interacting can be treated just as a special instance of the Markovian processes characterizing any single agent. Indeed, Friston and Frith demonstrate via computer simulation that when two birds equipped with active inference and precision-weighting sing to one another they necessarily exhibit generalized synchrony (Friston and Frith 2015b, Appendix A).

16 We note that this idea links directly to the idea that homeostatic information influences the overall precision of the brain. A neutral face will appear nice or nasty depending upon my prior sense of whether it will help me stay alive or not. We also note that generalized synchrony can be malicious; one agent can falsely express a state of happiness, in order to infect another agent with a false sense of understanding.

17 Cultural differences can also do this. For example, relative to European Americans, Asians show different neural processing in response to images of faces that represent a social-evaluative threat (Park and Kitayama 2012; also Xu et al. 2009). In-group members fail to understand out-group member actions—particularly prominent for out-groups who are disliked and dehumanized (Gutsell and Inzlicht 2010).

18 One reviewer rightly points out that the concept of Markov blanket is relative to how one defines the system. “There are blankets nested within blankets. When two people are interacting a blanket forms around them, but the individual blankets still remain (just as there is a blanket around an organism and around each of the cells of which the organism is composed). At an even higher level there are cultural Markov blankets maintaining the existence of learned societies and nations. This is a point that Chris Frith also has made in conversation. We agree.
